# Investigation Of Obesity-Related Mortality Rates In Delaware

**Published:** 2017-06

**Authors:** Malcolm J. D’Souza, Derald E. Wentzien, Riza C. Bautista, Catherine C. Gross

**Affiliations:** Wesley College, USA

**Keywords:** Delaware, Obesity, Mortality, SAS, GIS

## Abstract

As Delaware’s adult obesity crisis continues to be a leading public health concern, we evaluated Delaware’s 1999–2014 vital records to examine the association between obesity and mortality. We used the Delaware population death records from the Centers for Disease Control and Prevention (CDC) WONDER database and the Delaware Health Statistics Center (DHSC).

Together with the vital records, we incorporated Microsoft Excel, SAS (Statistical Analysis System) and GIS (geographic information system) tools to analyze obesity influences from county residence, economic status, education, gender, and race. Using the 15-year (1999–2014) time span with the CDC WONDER database, we observed a statistically significant 28.7% increase in the age-adjusted Delaware obesity-related mortality rates (where obesity was a contributory factor). Furthermore, obesity influenced death counts in all three Delaware counties (New Castle, Kent, and Sussex). Kent County experienced the largest increase (66.0%), followed by New Castle County (47.4%), and Sussex County (25.2%).

The DHSC mortality rates for all leading causes of death from 2000 to 2011 indicated relatively stable mortality rates for Delaware. However, using CDC WONDER data, the Delaware mortality rate for obesity as a single underlying cause in 2011 was 56.9% higher than mortality rate in 2000.

## INTRODUCTION

In the U.S. population, the weight-gain and resultant obesity epidemic has reached crisis proportions, and its public health impact (Dietz, 2015; Yang & Colditz, 2015) has had detrimental effects on the country’s gross national product (Malik, Willett, & Hu, 2013; Thornton & Beilfuss, 2016; Whaples, 2016). Globally, there is an enormous human burden of living with obesity as it relates to common health conditions, income, life expectancy, and mortality (Arnold et al., 2016; Prince et al., 2015; Rosengren et al., 2015; Stokes & Preston, 2016; Wadden, Brownell, & Foster, 2002; Wang, McPherson, Marsh, Gortmaker, & Brown, 2011; Ware, Gandek, & Allison, 2016). Furthermore, U.S. obesity disparities are found to be complex and dynamic but are proven to be associated with age, race, gender, income, racial/ethnic status, and geographic region divergence (Bower et al., 2015; Dinwiddie, Zambrana, Doamekpor, & Lopez, 2016; Shi, Zhang, van Meijgaard, MacLeod, & Fielding, 2015; Taber et al., 2012; Wang & Beydoun, 2007). From the global perspective of limited resources and budgets, innovative informatics technology systems can indeed provide the necessary solutions to tackle such human threats (Carayannis, Campbell, & Efthymiopoulos, 2014; Levi, Segal, St. Laurent, & Rayburn, 2014; Rosengren et al., 2015). Statistical Analysis System (SAS) software and geographic information system (GIS) tools serve as cutting-edge enabling technologies that help blend and bridge the big-data enigma in complex public health data sets (Alemayehu and Berger, 2016; Friede, Reid, & Ory, 1993; Raghupathi and Raghupathi, 2014).

In a 2015 GIS and SAS project (D’Souza, Kashmar, et al., 2015), undergraduates in our mentored research program (D’Souza, Kashmar, et al., 2015; D’Souza, Curran, Olsen, Nwogbaga, & Stotts, 2016; D’Souza & Wang, 2012; D’Souza, Kroen, Stephens, & Kashmar, 2015) analyzed available Delaware obesity-related data. We found (D’Souza, Kashmar, et al., 2015) an association between age and educational achievement and the state’s obesity prevalence. This study also showed that Delaware’s obesity progression rate was much greater in the less densely populated southern counties. The observed prevalent obesity trend was Kent County>Sussex County>New Castle County (D’Souza, Kashmar, et al., 2015).

A second Wesley College study determined undergraduate body mass index (BMI) using self-reported heights and weights (D’Souza, Walls, Rojas, Everett, & Wentzien, 2015). We found that 29.5% of Wesley’s student population (located in Kent County) was overweight and 19.8% was obese.

The two (D’Souza, Kashmar, et al., 2015; D’Souza, Walls, et al., 2015) project outcomes were not surprising. A prior foundation-supported public health report (Levi et al., 2014) documented an astronomical 113% rise in Delaware’s obesity rates from census year 1990 to 2014.

Starting with a 14.4% Delaware adult obesity rate in 1990 (Levi et al., 2014), the rate gradually increased to 17.1% in the next decennial census year, 2000. For the subsequent (2000–2010) decade, Delaware realized (Levi et al., 2014) an exponential 63.7% increase in its rates of adult obesity (to 28% in 2010). The following four years (to 2014) saw a 9.6% rise in obesity growth rate to 30.7%. In 2014, *The State of Obesity* (Levi et al., 2014) placed Delaware as having the 17th highest adult obesity rate in the nation.

This higher prevalence of obesity in Delaware (Levi et al., 2014; D’Souza, Kashmar, et al., 2015; D’Souza, Walls, et al., 2015) seems to be in alignment with the state’s geographic variations in the availability of fresh, affordable, local foods (Moss, 2015) and the enormous obesity-attributable medical expenditures (Finkelstein, Fiebelkorn, & Wang, 2004; Chang, Gertel-Rosenberg, Drayton, Schmidt, & Angalet, 2010) for chronic and disabling medical conditions (Chang et al., 2010; Gupta, 2014; Chang, Gertel-Rosenberg, & Snyder, 2014). Nationally, a number of studies have indicated strong associations between obesity and mortality risks (Stackelberg et al., 2012; Leung, Pollack, Colditz, & Chang, 2015; Baker et al., 2015; Masters et al., 2013; Kitahara et al., 2014; Finch & Crimmins, 2016). However, very little is known about how common behavioral risk factors and obesity contribute to mortality in Delaware (Flegal, Williamson, Pamuk, & Rosenberg, 2004).

To uncover obesity-related mortality disparities in Delaware, we utilized Microsoft Excel (Excel), GIS technology, and SAS programming to analyze (Cody & Smith, 1986; Venables & Ripley, 2013) the available mortality tables published by the CDC WONDER database (Friede et al., 1993) and the Delaware Vital Statistics Data, Delaware Health Statistics Center, Division of Public Health, Delaware Health and Social Services (Delaware Vital Statistics Data, 2015). In this study, data on race, socioeconomic status, age, sex, education, and geographic distributions were also obtained from other freely available state-by-state records and reports (Delaware Vital Statistics Data, 2015; U.S. Census Bureau, American FactFinder, 2015; Centers for Disease Control and Prevention, [CDC] 2015; U.S. Census Bureau, TIGER products, 2015; World Life Expectancy Rankings, 2016).

## MAPPING AND STATISTICAL METHODS

**Figure 1:**
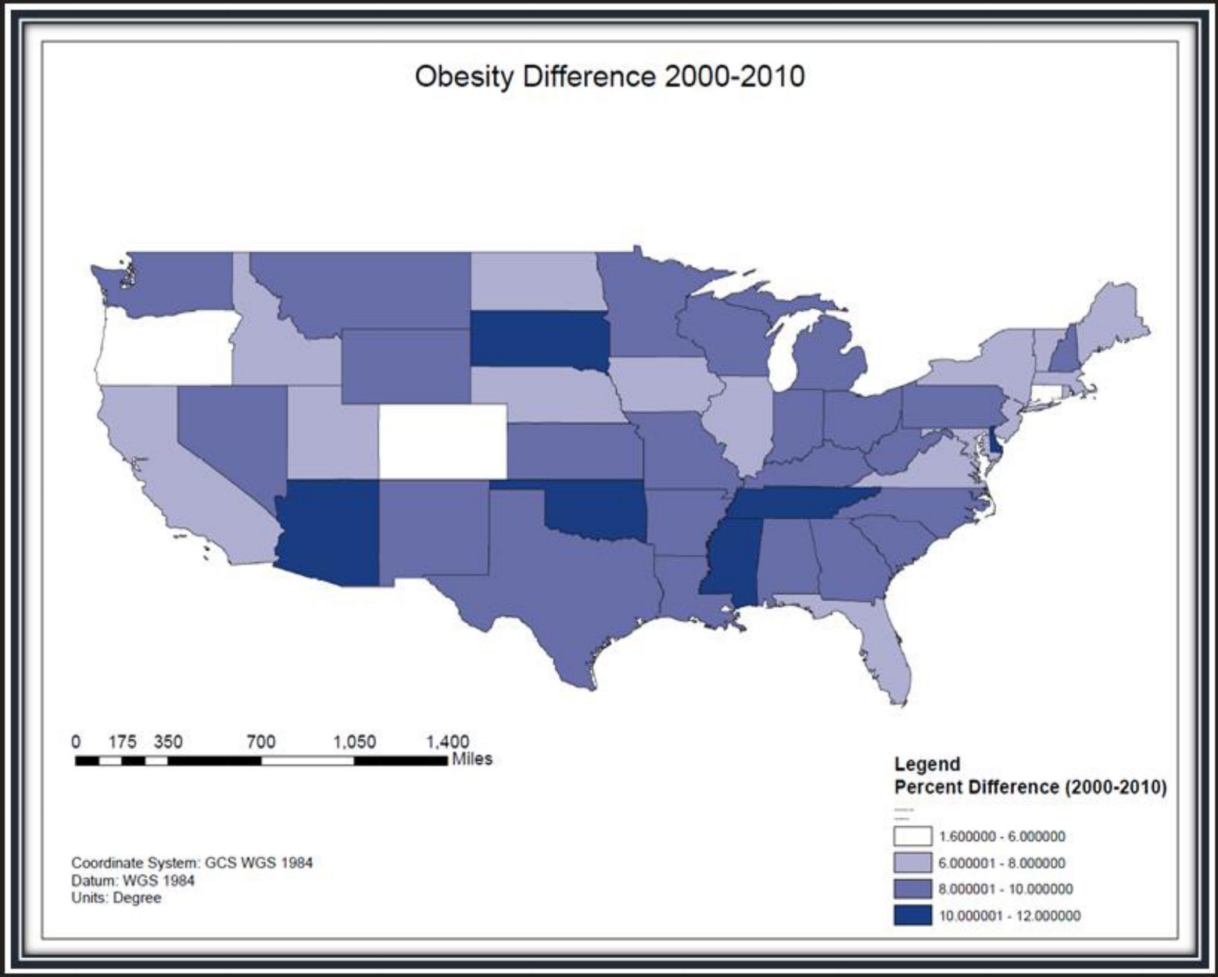
The GIS map shows that obesity rates in the United States have increased from 2000 to 2010. This map indicates that Delaware is one of six states placed in the highest obesity-difference classification.

GIS shapefiles of the 48 contiguous U.S. states were downloaded from the Topographically Integrated Geographic Encoding and Referencing (TIGER) files (U.S. Census Bureau, 2015) available from the U.S. Census Bureau and imported into the ArcGIS 10.3 software (ESRI). For each of the 48 contiguous U.S. states annual obesity rates for 2000–2010 were obtained from the Centers for Disease Control and Prevention and the U.S. Census Bureau (American FactFinder, 2015; CDC, 2015). Figure 1 shows a color-gradated map of the 48 states depicting their obesity growth rate changes from 2000 to 2010.

The 2000 U.S. census reported that Delaware’s population was 783,596, and in 2010, census records indicate that the population grew by 14.6% to 897,934 (U.S. Census Bureau, American FactFinder, 2015). In Figure 1, the percentage differences for the obesity-growth trend comparison reveals the presence of massive percentage changes occurring in the six states that are shown with the darkest color grades: Arizona (70.5%), Delaware (63.7%), Oklahoma (56.2%), South Dakota (56.0%), Tennessee (52.6%), and Mississippi (45.1%).

During the first decade of the 21st century, Figure 1 data demonstrate that the First State obesity rates grew the second fastest in the nation, with an annual obesity growth rate of 6.4%. Accordingly, *The State of Obesity* (Levi et al., 2014) ranking of the highest obesity rates showed Delaware’s state rank bouncing up from 37 in 2000 to 21 in 2010.

The mortality research and analyses for this article used data from two sources: the Centers for Disease Control and Prevention (CDC) WONDER data (Friede et al., 1993) and the Delaware Health Statistics Center (Delaware Vital Statistics Data, 2015). Statewide 1999–2014 obesity-related mortality statistics were obtained from the CDC menu-driven query WONDER data (Friede et al., 1993). The CDC WONDER 1999–2014 data identify the underlying cause of death and demographic information for U.S. residents (Friede et al., 1993). The advantage of this CDC data set is that there is an option to request 95% confidence levels for both crude and age-adjusted (weighted averages of the age-specific death rates, where the weights represent a fixed population by age) mortality rates. In addition, for each of the three Delaware’s counties, we also obtained the complete 2000–2011 DHSC annual data set containing one record on each deceased person for each year stratified by the underlying cause of death (Delaware Vital Statistics Data, 2015; U.S. Census Bureau, American FactFinder, 2015).

For the 1999–2014 and the 2000–2011 Delaware data records, we utilized the Excel platform and SAS programming to data-mine the CDC WONDER (Friede et al., 1993) and the DHSC mortality records (Delaware Vital Statistics Data, 2015). Tables and graphs were first created using Excel and then SAS techniques were used to create SAS data lines and JPG files. This allowed us to evaluate, analyze, and report any mortality-related trends (and/or impacts) that could be correlated to various parameters, including diseases linked to obesity (Dietz, 2015; Yang & Colditz, 2015; Malik et al., 2013; Thornton & Beilfuss, 2016; Stokes & Preston, 2016; Wadden et al., 2002; Wang et al., 2011, Prince et al., 2015; Ware et al., 2016; Arnold et al., 2015; Rosengren et al., 2015; Taber et al., 2012; Shi et al., 2015; Finkelstein et al., 2004; Chang et al., 2010, 2014; Gupta, 2014; Masters et al., 2013; Kitahara et al., 2014; Finch & Crimmins, 2016; Flegal et al., 2004; Delaware Vital Statistics Data, 2015; U.S. Census Bureau, American FactFinder, 2015; CDC, 2015), socioeconomic factors (based on county residence, income, education) (Dietz, 2015; Yang & Colditz, 2015; Malik et al., 2013; Thornton & Beilfuss, 2016; Whaples, 2016; Stokes & Preston, 2016; Wadden et al., 2002; Wang et al., 2011; Arnold et al., 2015; Rosengren et al., 2015; Wang & Beydoun, 2007; Dinwiddie et al., 2016; Taber et al., 2012; Bower et al., 2015; Shi et al., 2015; Levi et al., 2014; Friede et al., 1993; Alemayehu & Berger, 2016; Raghupathi & Raghupathi, 2014; D’Souza, Walls, et al., 2015; Moss, 2015; Finkelstein et al., 2004; Chang et al., 2010, 2014; Gupta, 2014; Masters et al., 2013; Kitahara et al., 2014; Finch & Crimmins, 2016; Flegal et al., 2004; Delaware Vital Statistics Data, 2015; U.S. Census Bureau, American FactFinder, 2015; CDC, 2015), age (Dietz, 2015; Yang & Colditz, 2015; Malik et al., 2013; Thornton & Beilfuss, 2016; Whaples, 2016; Stokes & Preston, 2016; Wadden et al., 2002; Wang et al., 2011; Prince et al., 2015; Ware et al., 2016; Arnold et al., 2015; Rosengren et al., 2015; Wang & Beydoun, 2007; Taber et al., 2012; Bower et al., 2015; Shi et al., 2015; Friede et al., 1993; Finkelstein et al., 2004; Chang et al., 2010, 2014; Gupta, 2014; Masters et al., 2013; Kitahara et al., 2014; Finch & Crimmins, 2016; Delaware Vital Statistics Data, 2015; U.S. Census Bureau, American FactFinder, 2015; CDC, 2015), gender, and race (Dietz, 2015; Yang & Colditz, 2015; Malik et al., 2013; Thornton & Beilfuss, 2016; Wang et al., 2011, Prince et al., 2015; Ware et al., 2016; Arnold et al., 2015; Rosengren et al., 2015; Wang & Beydoun, 2007; Dinwiddie et al., 2016; Taber et al., 2012; Shi et al., 2015; Friede et al., 1993; Alemayehu & Berger, 2016; Raghupathi & Raghupathi, 2014; Finkelstein et al., 2004; Chang et al., 2010, 2014; Gupta, 2014; Masters et al., 2013; Kitahara et al., 2014; Finch & Crimmins, 2016; Flegal et al., 2004; Delaware Vital Statistics Data, 2015; U.S. Census Bureau, American FactFinder, 2015; CDC, 2015; U.S. Census Bureau, TIGER products, 2015).

The CDC WONDER is a query-based database that provided Delaware obesity-related mortality information from 1994 to 2014 with 95% confidence interval limits and age-adjusted mortality rates with 95% confidence intervals. Drop down menus were used to further filter the data based on location, age, gender, race, place of death, and ICD-10 codes (universal medical diagnosis codes). The age category options (<1, 1–4, 5–9, 10–14, 15–19, 20–24, 25–34, 35–44, 45–54, 55–64, 65–74, 75+) matched those recorded in the DHSC data. Gender menu options included female and male. Race menu options included American Indian or Alaska Native, Asian or Pacific Islander, Black or African American, and White. The place of death menu options included medical facility–inpatient, medical facility–outpatient or emergency room, medical facility–dead on arrival, medical facility–status unknown, decedent’s home, hospice facility, nursing home/long-term care, other, place of death unknown. To identify trends for obesity as the single underlying cause of death (UCD), ICD-10 Codes E66 identified overweight and obesity and the multiple causes of death (MCD) selection tab was left blank. On the other hand, to obtain information for any mention of obesity on the death record, the ICD-10 codes ALL (all causes of death) tab was selected and under the MCD-ICD-10 Codes, the E66 code tab was selected.

The DHSC website (Delaware Vital Statistics Data, 2015) contained (complete) annual individual mortality information for Delaware from 2000 to 2011. The variables recorded in the DHSC website included the year of death, residence information, leading cause of death, place of death, and demographic information.

The variables in the DHSC files permitted analysis on Delaware mortality rates based on residency, gender, race, age, and education. The year of death, state, county, gender, education level, and race were used in their current format. The year of death values recorded the year of the decedent’s death. The decedent’s residency was based on the Federal Information Processing Standard (FIPS) state codes. The decedent’s county of residency was recorded as 1: Kent County, 3: New Castle County, 5: Sussex County, 777: Out of State, 999: Unknown. Gender was recorded as 1: Male, 2: Female, 9: Unknown. The education level was recorded as 1: <12 years, 2: 12 years, 3: 13+ years, 9: Unknown. The DHSC files helped us to compile and calculate the 2000–2011 Delaware mortality rates.

## RESULTS AND DISCUSSION

When using the 1999–2014 CDC WONDER database (Friede et al., 1993) mortality rates where obesity was listed as the single UCD, no statistically significant differences were found, since the mortality rates for some years were listed in CDC WONDER as being unreliable. Delaware is a small state and the number of such obesity-related deaths (where obesity is the single UCD) was too small for statistical testing. However, the corresponding single UCD rates (deaths/population) were calculated and line graphs constructed to visualize any trend.

**Figure 2:**
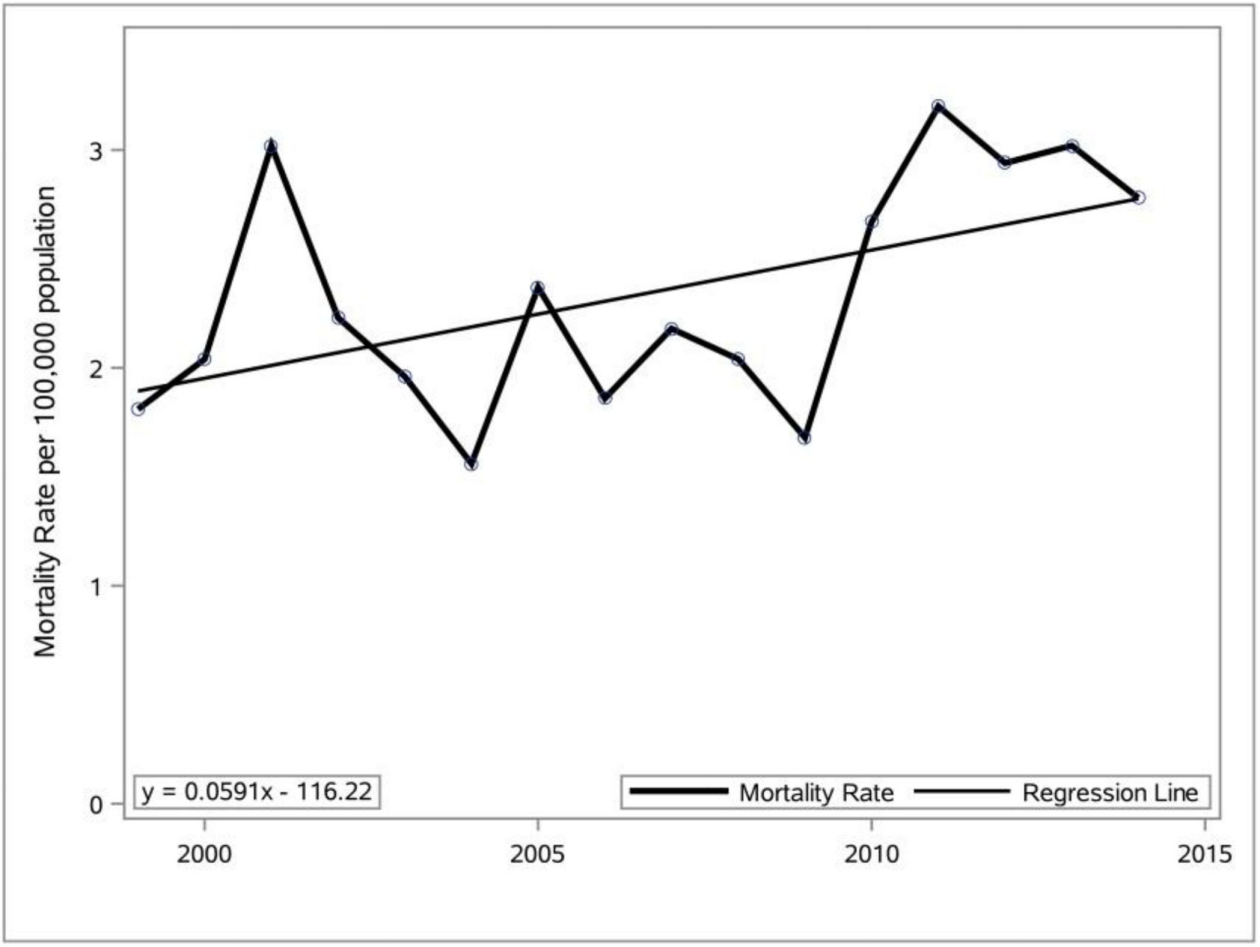
Percentage change (1999–2014) in Delaware mortality rate where obesity was listed as the single UCD. Data source: CDC WONDER vital records.

The Delaware mortality crude rate per 100,000 (population) for obesity as the single UCD on the death certificate from 1999 to 2014 for individuals over the age of 15 years was data-mined using SAS and was used to prepare the line graph and linear equation presented in Figure 2.

Analysis of the Figure 2 data indicates that the crude Delaware obesity mortality rate (deaths/population) for individuals over the age of 15 was 1.81 in 1999, and it increased to 2.78 in 2014. This indicates that the 2014 crude Delaware obesity mortality rate (where obesity is the single UCD) was 53.6% higher than the 1999 crude obesity mortality rate.

**Figure 3:**
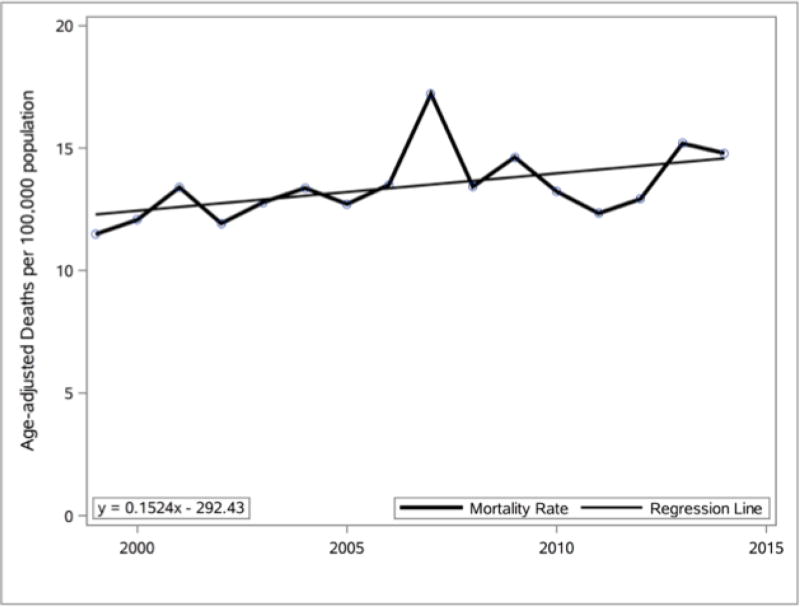
Percentage change (1999–2014) in Delaware age-adjusted mortality rate for any mention of obesity on death certificate. Data source: CDC WONDER vital records.

The age-adjusted mortality rate (Rosengren et al., 2015) is a weighted average of the age-specific death rates, where the weights represent a fixed population by age. From 1994 to 2014, for individuals over the age of 15, the Delaware age-adjusted mortality rate for any mention of obesity on the death certificate was data-mined and was used within a SAS data set to prepare the line graph and linear equation presented in Figure 3. Analysis of the Figure 3 data indicates that the Delaware age-adjusted obesity mortality rate was 11.49 (95% confidence interval [CI], 8.98–14.49) in 1999 and it increased to 14.79 (95% CI, 12.11–17.48) in 2014. This indicates that the 2014 age-adjusted obesity mortality rate was 28.7% higher than the 1999 age-adjusted obesity mortality rate in Delaware.

**Figure 4:**
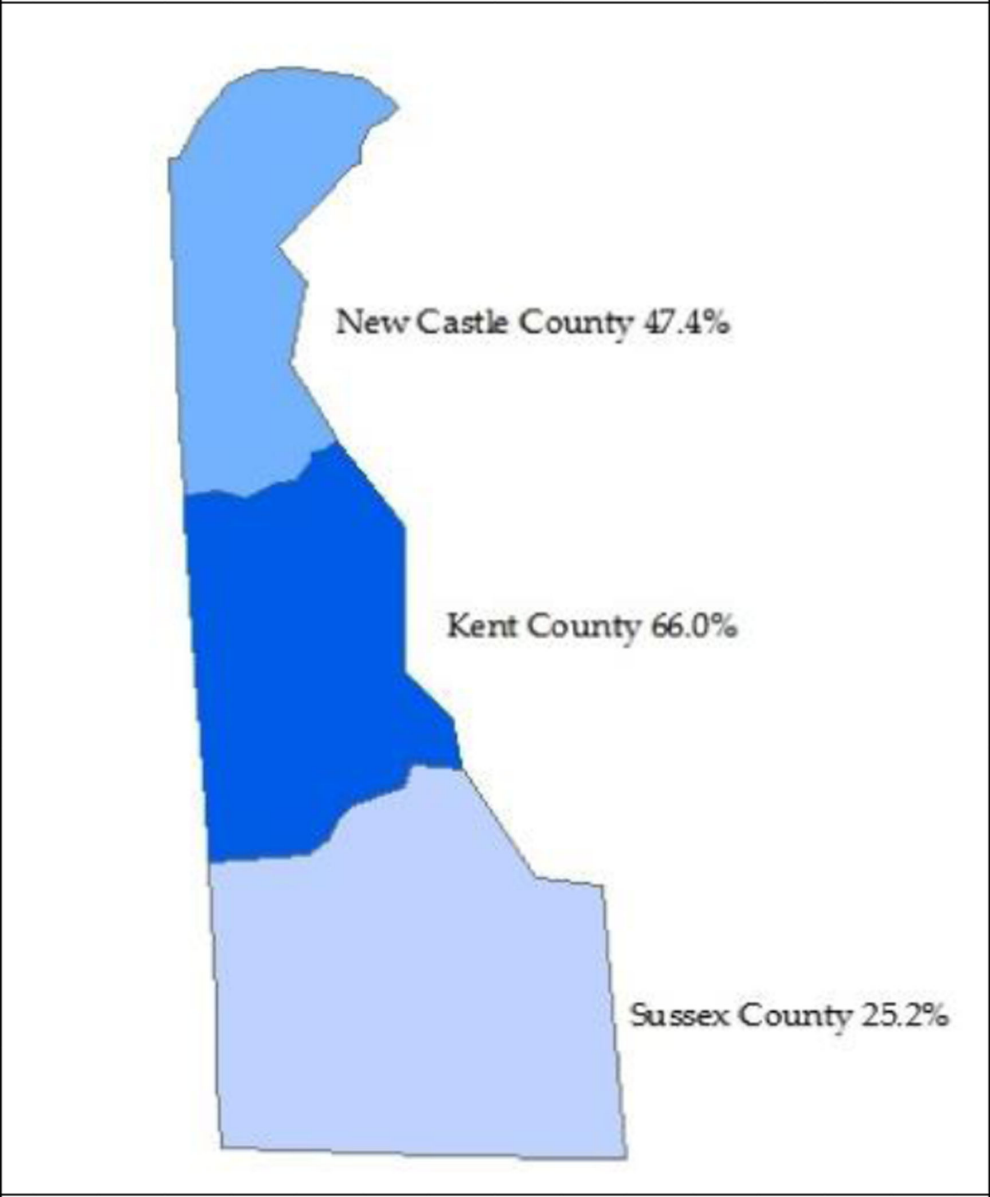
This chloropleth GIS map details change in the obesity mortality rates for Delaware’s three counties from 1999 to 2014. The color gradients show that the highest rate is in Kent County. Data source: CDC WONDER vital records.

The Delaware obesity-related mortality rates for any mention of obesity on the death certificate were calculated for the three counties (New Castle, Kent, and Sussex) from 1999 to 2014. The changes in the obesity mortality rates for the three counties from 1999 to 2014 were calculated and GIS was used to generate the information presented in Figure 4. All three Delaware counties had an increase in the obesity mortality rate. Kent County experienced the largest increase (66.0%), followed by New Castle County (47.4%), and Sussex County (25.2%).

The individual records in the DHSC data sets (Delaware Vital Statistics Data, 2015) were used to calculate the total mortality rates in Delaware and the three counties (New Castle, Kent, and Sussex) for individuals over the age of 15 years minus deaths due to motor-vehicle and non-transport accidents, suicides, and homicides (as these were not specified as being health-related). The mortality rates per 100,000 for each year were calculated using Equation 1.

(1) $$\text{Mortality rate for year }i=\frac{\text{number of deaths in year }i}{\text{population in year }i}\cdot 100,000$$

**Figure 5:**
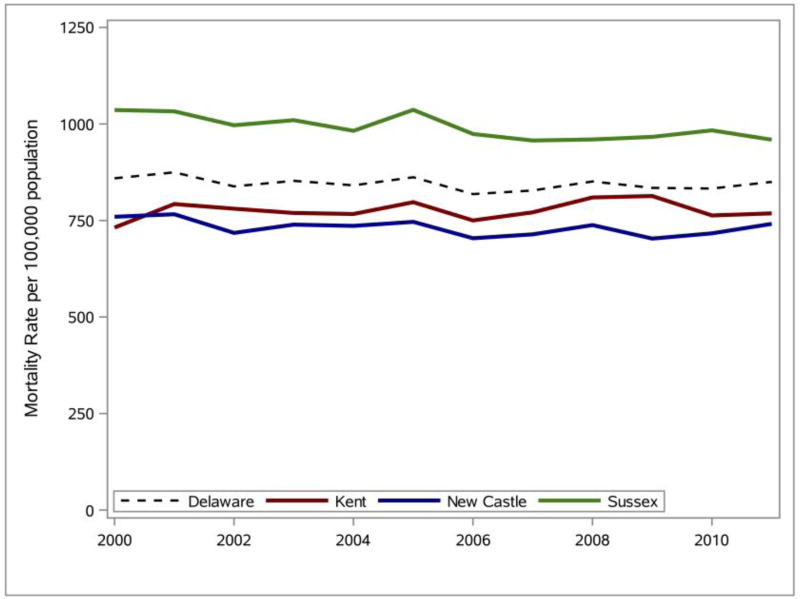
Percentage change (2000–2011) in Delaware mortality rate for all leading causes of death. Data source: DHSC (Delaware Vital Statistics Data, 2015).

The mortality rates for all leading causes of death (excluding those due to motor-vehicle and non-transport accidents, suicides, and homicides) in Delaware from 2000 to 2011 were calculated and SAS was used to generate the line graphs presented in Figure 5. Analysis of the line graphs indicates relatively stable mortality rates for Delaware and each of the three counties between 2000 and 2011. The mortality rate for Delaware in 2011 is 1.07% lower than the mortality rate in 2000. The change in the mortality rate for the three counties over the same time period is New Castle: +5.03%, Kent: −2.42%, and Sussex: −7.42%.

**Figure 6:**
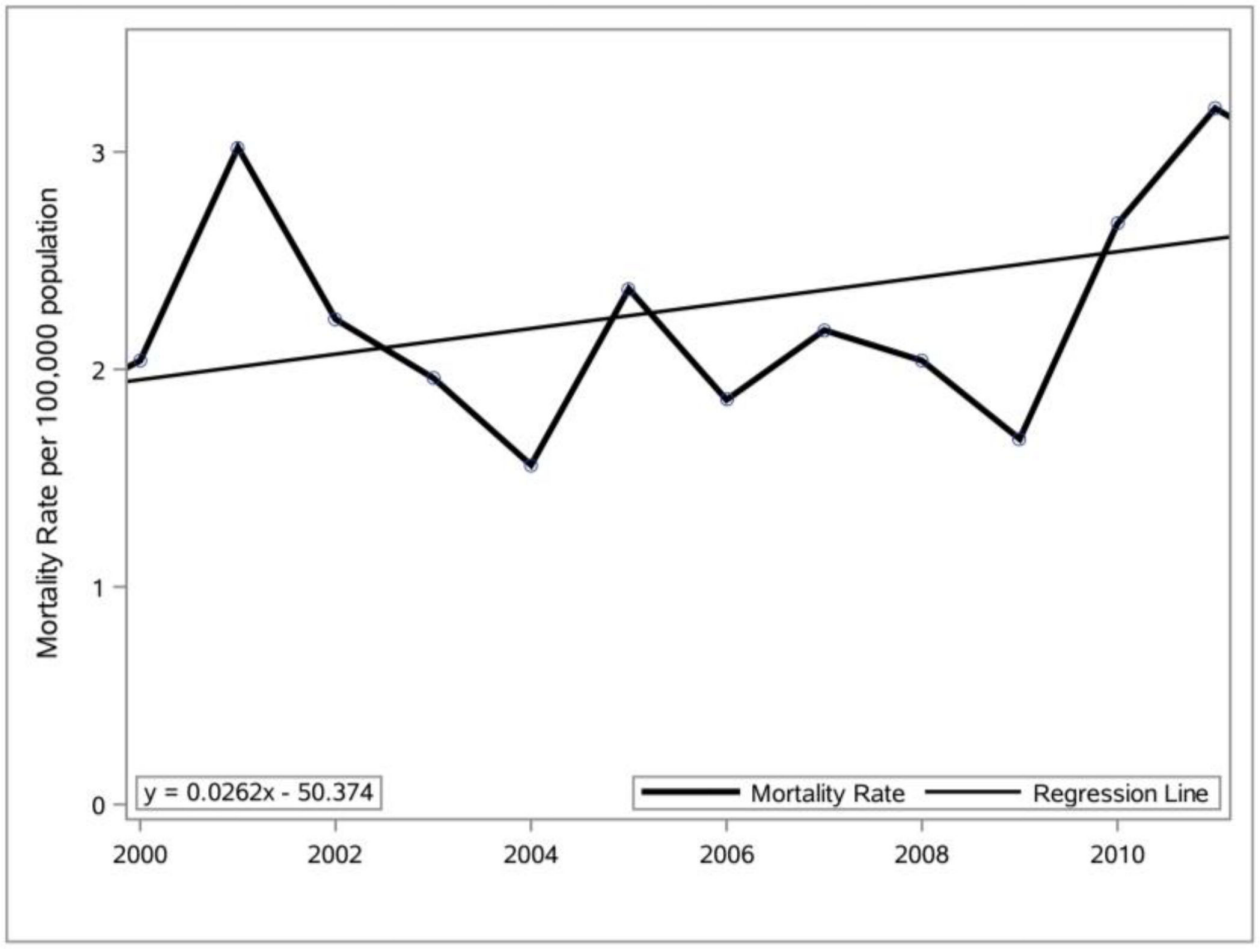
Percentage change (2000–2011) in mortality rate (Friede et al., 1993) for Delaware where obesity was listed as the single UCD. Data source: CDC WONDER vital records.

The Delaware mortality rate for obesity as the single UCD from 2000 to 2011 for individuals over the age of 15 was used in SAS to prepare the line graph and linear equation presented in Figure 6. The Delaware mortality rates (Figure 5) were then compared to the Delaware obesity mortality rates (Figure 6). The Delaware mortality rate for obesity as a single underlying cause in 2011 was 56.9% higher than the mortality rate in 2000, which is very much greater than the 1.07% decrease in the Delaware mortality rate over the same time period. Further analysis of the two graphs indicates that the Delaware mortality rate remained relatively flat while the Delaware obesity mortality rate showed a significant increase in the last two years analyzed.

**Figure 7:**
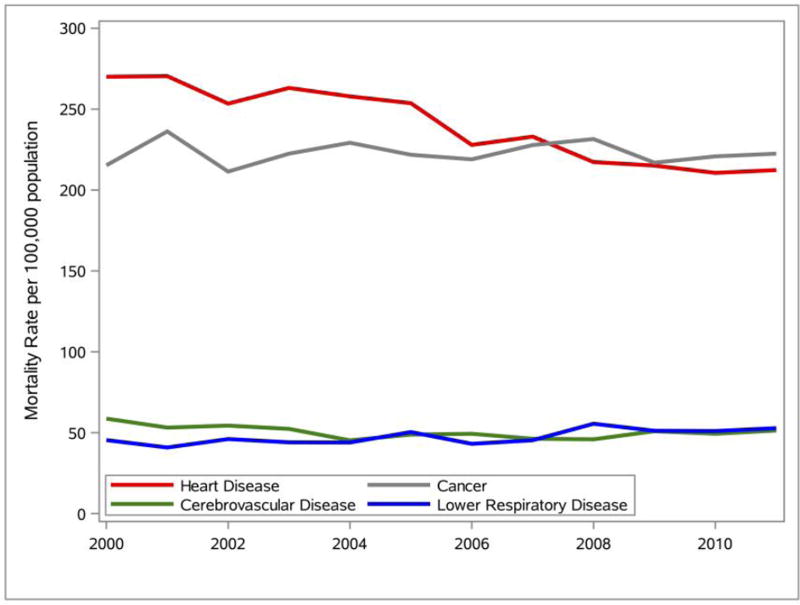
The Delaware mortality rate for the top four leading causes of death (2000–2011). Data source: DHSC (Delaware Vital Statistics Data, 2015).

The Delaware mortality rates for the top four leading causes of death from 2000 to 2011 were calculated and SAS was used to generate the line graphs presented in Figure 7. Analyses of the line graphs indicate that heart disease decreased by 21.36%, cancer increased by a modest 3.34%, cerebrovascular disease decreased by 12.47%, and lower respiratory disease increased by 16.25%. The graph also indicates that although heart disease was the leading cause of death in Delaware from 2000 to 2011, cancer had the highest mortality rate for Delaware in 2011.

A Pearson correlation test was performed on the county mortality rates (Delaware Vital Statistics Data, 2015) and each of three socioeconomic variables (poverty, income, and education attainment) using the PROC CORR procedure in SAS. The R^2^ and *p* values are listed in Table 1. No significant correlations were found between the county mortality rates and the poverty or the education levels. No significant correlations were found between the county mortality rates and per-capita income levels, except for Sussex County, which was very close to a 5% threshold (*p* = .0513).

Table 2 shows the 2010 demographics, social characteristics, economic profile, and education level (>25 years of age and with a bachelor’s degree) of Delaware’s adult population in each of the three Delaware counties. The calculated mortality rate is based on any mention of obesity on a death certificate.

**Figure 8:**
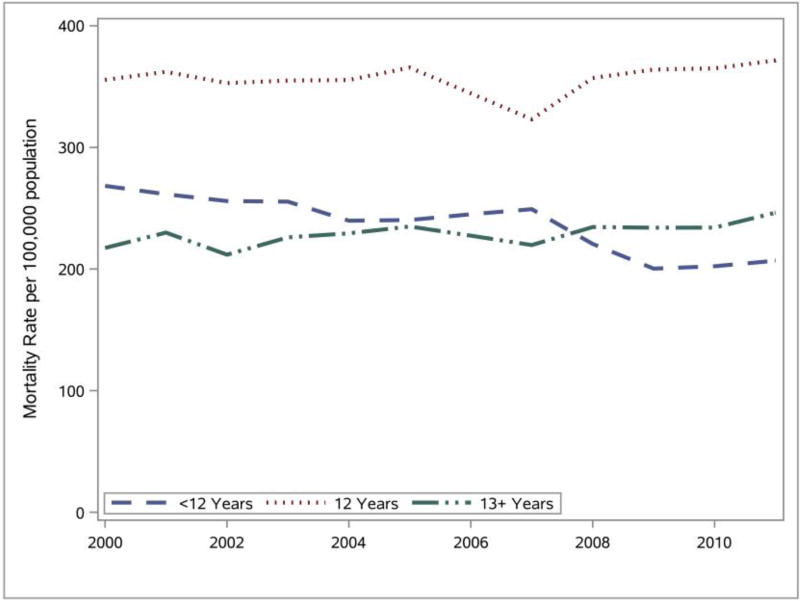
The Delaware mortality rate (2000–2011) for all leading causes of death by education level. Data source: DHSC (Delaware Vital Statistics Data, 2015).

The Delaware mortality rates for three education levels (<12 years, 12 years, 13+ years) were calculated and SAS was used to generate the line graphs presented in Figure 8. In addition, due to a discrepancy in the DHSC 2006 educational attainment data, the method of polynomial (1st degree) interpolation (Cody & Smith, 1986; Venables & Ripley, 2013; Gentle, Härdle & Mori, 2012) was used to estimate the deceased’s educational attainment for that year. Analyses of the line graphs indicate that those with a high school diploma (12 years) consistently had the highest mortality rate. Individuals with less than a high school diploma and with more than a high school diploma had very similar mortality rates. Further analyses indicate that the Delaware mortality rates remained relatively stable for each of the education levels between 2000 and 2011.

**Figure 9:**
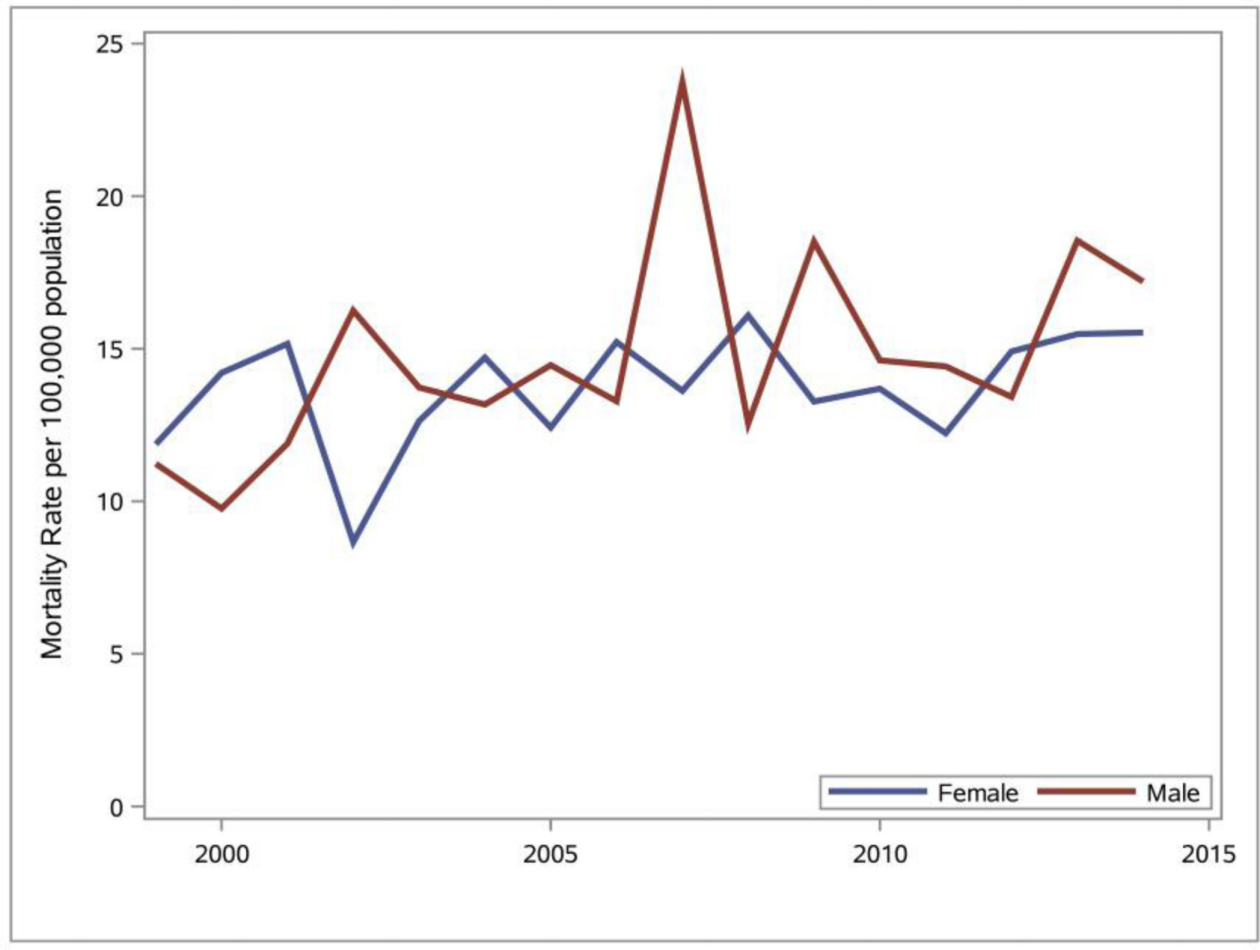
Gender differences observed for any mention of obesity on death certificate (Friede et al., 1993) in Delaware, 1999–2014. Data source: DHSC (Delaware Vital Statistics Data, 2015).

The Delaware obesity mortality rates for females and males were calculated and SAS was used to generate the line graphs presented in Figure 9. Analyses of the line graphs indicate that there is not a distinguishable difference in the obesity mortality rates between males and females between 1999 and 2014 except in 2006, when males had a higher mortality rate.

**Figure 10:**
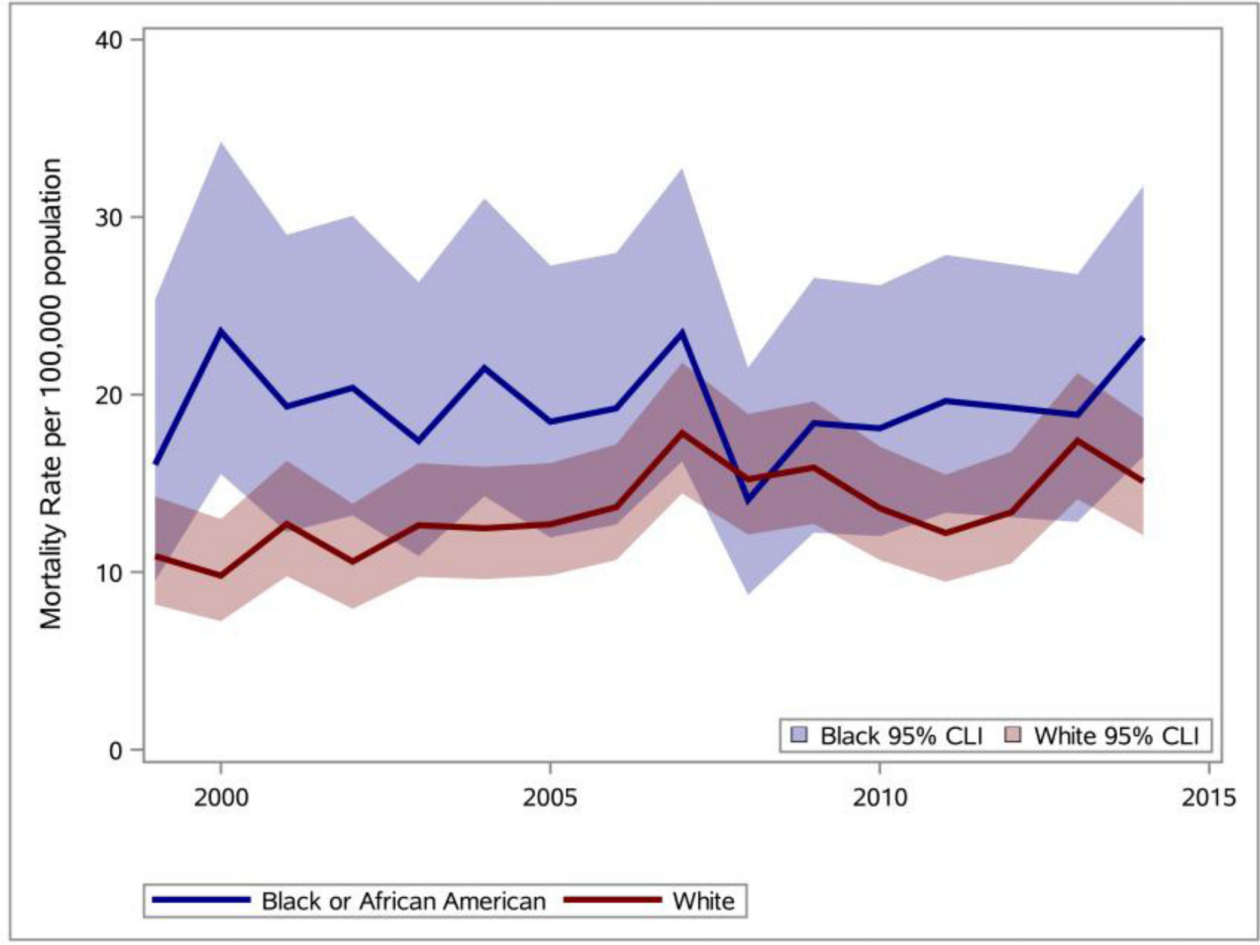
Racial disparities (Black or White) observed for any mention of obesity on death certificate (Friede et al., 1993) in Delaware (1999–2014). Data source: DHSC (Delaware Vital Statistics Data, 2015).

The Delaware obesity mortality rates for Black or African American and White were calculated and SAS was used to generate the line graphs presented in Figure 10. Analyses of the line graphs indicate that the Black or African American obesity mortality rates (solid blue line) in all years except one were greater than the White obesity mortality rates (solid red line). 95% confidence intervals for Black or African American obesity mortality rates and White obesity mortality rates are also shown. Complete separation is not observed between the 95% confidence intervals for the Black or African American obesity mortality rates and the White obesity mortality rates.

## CONCLUSIONS

The Delaware age-adjusted mortality rate attributable to obesity (where there was any mention of obesity on the death certificate) for individuals over the age of 15 in 2014 was 28.7% higher than the rate observed in 1999, and except for one year, the obesity-related death rate for Blacks exceeded that for Whites. Furthermore, all three Delaware counties (New Castle, Kent, and Sussex) saw an increase in death counts influenced by obesity.

The DHSC 2000–2011 vital records showed that even though mortality rates due to the top three causes of death steadily declined in Delaware, the mortality rate due to obesity as the single UCD increased by 56.9% during this time-frame. For Sussex County, the relationship between obesity-related mortality causes and per-capita income was close to being statistically significant (5.13%).

## Figures and Tables

**Table 1:** Correlation test results (R^2^ and *p* values) between mortality rates (per 100,000) in the three Delaware counties and each of the three socioeconomic variables (poverty, income, and educational attainment) for the time-series 2000–2010.

	New Castle	Kent	Sussex
Poverty			
R^2^	.3342	.0094	.3134
*p*	.1739	.8362	.1913
Per-Capita Income			
R^2^	.0819	.2460	.3594
*p*	.3936	.1208	.0513
Educational Attainment			
R^2^	.0379	.0007	.4013
*p*	.6757	.9563	.1267

Data source: DHSC (Delaware Vital Statistics Data, 2015).

**Table 2:** 2010 demographics, social characteristics, economic profile, and education level (>25 years of age and with a bachelor’s degree) of Delaware’s adult population in each of the three Delaware counties. The calculated mortality rate is based on any mention of obesity on death certificate from CDC WONDER for 2010 (Friede et al., 1993).

Delaware County	Mortality Rate per 100,000	Per-Capita Income ($)	Educational Attainment	Poverty
New Castle	12.63	44920	19.6%	11.3%
Kent	11.09	34344	12.5%	11.2%
Sussex	8.62	35666	11.7%	13.8%
